# Induction chemotherapy for the individualised treatment of hypopharyngeal carcinoma with cervical oesophageal invasion: a retrospective cohort study

**DOI:** 10.1186/s12957-020-02095-0

**Published:** 2020-12-11

**Authors:** Tian-Qiao Huang, Ru Wang, Ju-Gao Fang, Shi-Zhi He, Qi Zhong, Li-Zhen Hou, Hong-Zhi Ma, Xiao-Hong Chen, Xue-Jun Chen, Ping-Dong Li, Ling Feng, Qian Shi, Meng Lian

**Affiliations:** Department of Otolaryngology Head and Neck Surgery, Beijing Tongren Hospital, affiliated with Capital Medical University, Beijing, 100730 China

**Keywords:** Hypopharyngeal carcinoma, Induction chemotherapy, Oesophageal invasion, Overall survival, Personalised medicine

## Abstract

**Background:**

This study aimed to evaluate the potential of induction chemotherapy as an indicator of the management of advanced hypopharyngeal carcinoma with cervical oesophageal invasion.

**Methods:**

Sixty-eight patients admitted to our hospital between February 2003 and November 2016 with stage IVB hypopharyngeal carcinoma with cervical oesophageal invasion were retrospectively analysed. Patients were divided into two groups according to the treatment they selected following an explanation of the different treatments available. Patients in group A received induction chemotherapy and had (1) complete/partial remission following chemotherapy and radiotherapy/concurrent chemoradiotherapy or (2) stable disease following chemotherapy and surgery. Patients in group B underwent surgery followed by adjuvant radiotherapy/concurrent chemoradiotherapy. Survival analyses were performed using the Kaplan–Meier method, and differences between the groups were evaluated using the log-rank test. Laryngeal and oesophageal retention rates were compared using the cross-tabulation test.

**Results:**

The 3- and 5-year overall survival rates were 22.86% and 11.43% in group A and 24.25% and 6.06% in group B, respectively (all *P* > 0.05). The laryngeal and oesophageal retention rates were 40.0% and 74.3% in group A and 0.0% and 27.3% in group B, respectively (all *P <* 0.01). There was no statistically significant difference in the incidence of post-operative complications between the two groups (group A 8.6%, group B 12.1%; *P* > 0.05).

**Conclusions:**

Induction chemotherapy may be an appropriate first choice to ensure laryngeal and oesophageal preservation in the individualised treatment of advanced hypopharyngeal carcinoma with cervical oesophageal invasion.

## Background

Hypopharyngeal carcinoma is one of the most malignant head and neck carcinomas and is associated with a poor prognosis. Although the survival of patients has improved over the past three decades, the 5-year overall survival (OS) rate is only approximately 40% [1, 2]. The National Comprehensive Cancer Network guidelines [3] recommend surgical and non-surgical approaches as effective treatment strategies for advanced hypopharyngeal carcinoma. Recommended treatment strategies include radiotherapy, concurrent chemoradiotherapy, and surgery based on the curative effect of induction chemotherapy.

Induction chemotherapy, or neoadjuvant chemotherapy as it is also known, has been used to treat head and neck carcinoma in recent years, including hypopharyngeal carcinoma. In the short-term, it has been shown to reduce tumour volume and maximise the retention of organ function [4]. The function of the pharynx, larynx, trachea, and oesophagus should be considered in the management of advanced hypopharyngeal carcinoma with cervical oesophageal invasion. Several studies [5, 6] have investigated the treatment of hypopharyngeal and oesophageal carcinoma. However, there is little experience in the treatment of advanced hypopharyngeal carcinoma with cervical oesophageal invasion. Furthermore, few studies have reported on patients screened during induction chemotherapy who received individualised treatment. In this study, we retrospectively analysed the clinical data of advanced hypopharyngeal carcinoma patients with cervical oesophageal invasion. The therapeutic effects of two treatment modalities were compared to provide a reference for selecting the appropriate treatment strategy for advanced hypopharyngeal carcinoma with cervical oesophageal invasion.

## Methods

### Patients

Sixty-eight hypopharyngeal carcinoma patients with cervical oesophageal invasion who were admitted to our hospital between February 2003 and November 2016 were enrolled. Complete follow-up data were retrospectively reviewed. The age of the patients ranged from 36 to 77 years, and the median age was 54.5 years. Sixty-three patients were men, and five were women. Among all patients, 14 had high pathological differentiation, 35 had moderate differentiation, and 19 had poor differentiation. The primary lesion was located in the pyriform sinus in 27 patients, posterior pharyngeal wall in 23 patients, and post-cricoid region in 18 patients. Gastroscopy and enhanced neck and chest computed tomography (CT; oesophageal barium meal) were used to identify cervical oesophageal invasion prior to treatment. According to the American Joint Committee on Cancer, seventh edition (2010), clinical physical examination, and pre-treatment imaging, the clinical stage was determined to be stage IVB for all patients.

### Treatment groups

Patients were divided into two groups according to the treatment modality. Surgery was the treatment modality of choice before 2010. After 2010, patients were informed of the different treatment options available and grouped according to their preference. Thirty-five patients selected induction chemotherapy (group A). All of the patients in group A had (1) a complete/partial response (CR/PR) following chemotherapy and radiotherapy/concurrent chemoradiotherapy or (2) stable disease (SD) following chemotherapy and surgery. Thirty-three patients underwent surgery and post-operative adjuvant radiotherapy/concurrent chemoradiotherapy (group B). Patients underwent surgery of complete resection of the lesion, and the incision edge was examined for rapid frozen pathological examination during the operation, so as to ensure complete resection of the tumour at a micro or macro level. There were no differences in baseline clinical characteristics between the two groups (Table 1).

**Table 1:** Patient characteristics

Characteristic	Classification	Total (*N* = 68)	Group A (*N* = 35)	Group B (*N* = 33)	*χ*^2^ value	*P* value
Sex	Male	63	33	30	0.2843	0.5939
	Female	5	2	3		
Age (years)	≤ 60	50	26	24	0.1675	0.8970
	> 60	18	9	9		
Subsite	Pyriform sinus	27	14	13	0.0217	0.9892
	Post-cricoid region	23	12	11		
	Posterior wall	18	9	9		
Differentiation	High	14	8	6	0.3084	0.8571
	Moderate	35	18	17		
	Poor	19	9	10		
N classification	N0	19	8	11	0.4786	0.4890
	N1–3	49	27	22		
Drinking	Yes	42	22	20	0.0035	0.9532
	No	26	13	13		
Smoking	Yes	47	26	21	0.4725	0.4918
	No	21	9	12		

### Treatment regimens

The induction chemotherapy regimen for patients in group A was two 21-day cycles of paclitaxel, platinum, and fluorouracil (TPF) [6, 7]. Two weeks after the completion of induction chemotherapy, the outcomes were evaluated using enhanced neck CT, stroboscopic laryngoscopy, and in some cases biopsy. Patients with CR (complete remission of the primary tumour; *N* = 4) or PR (shrinkage of the primary tumour by ≥ 70%; *N* = 9) received concurrent chemoradiotherapy/radiotherapy after induction chemotherapy. Patients with SD (no change or shrinkage of the primary tumour by < 70%; *N* = 22) were treated surgically after chemotherapy. Chemotherapeutic responses were evaluated after cycles of TPF using the revised Response Evaluation Criteria in Solid Tumors (version 1.1) [8]. Tumour volume and change in volume were based on the tumour length and width assessed by enhanced neck CT. In detail, all the diameters of target lesions including both primary lesions and measurable lymph nodes no shorter than 15 mm in short axis were measured. The criterion of CR is disappearance of all target lesions. Any pathological lymph nodes (whether target or nontarget) must have reduction in short axis to < 10 mm. The criterion of PR is at least a 70% decrease in the sum of diameters of target lesions. The criterion of SD is shrinkage less than 30% or increase less than 20%. One patient with SD underwent transoral laser resection of hypopharyngeal carcinoma, preserving the larynx, hypopharynx, and oesophagus. Twelve patients underwent total laryngectomy and partial hypopharyngeal resection, preserving the oesophagus. Nine patients underwent total laryngectomy and oesophageal resection.

Group B comprised 33 patients. Nineteen patients underwent complete laryngeal and oesophageal resection and gastric pharyngostomy. Six patients underwent partial pharyngeal and oesophageal resection and residual larynx-gastric anastomosis. Four patients underwent complete laryngeal resection with partial pharyngeal and oesophageal resection and pectoralis major musculocutaneous flap repair. Four patients underwent complete laryngeal and oesophageal resection and repair using a free jejunum/ileum flap. All patients underwent simultaneous neck dissection and post-operative radiotherapy/concurrent chemoradiotherapy within 6 weeks. Twenty-one patients received radiotherapy (65–70 Gy) alone, and 12 patients underwent concurrent chemoradiotherapy (platinum plus paclitaxel).

In total, 55 patients underwent R1/R2 resection, including 22 patients in group A and 33 patients in group B.

### Follow-up

Patients were followed-up by outpatient review and telephone until November 2019. Three patients were alive at the end of follow-up (the remaining 65 patients had died).

### Statistical analyses

OS was defined as the time from the date of surgery or treatment initiation to the date of death or last follow-up. Survival analyses were performed using the Kaplan–Meier method, and differences between the two groups were evaluated using the log-rank test. Laryngeal and oesophageal retention rates were compared using the cross-tabulation method. Categorical variables were compared using the chi-square test (SPSS 22.0, *P* value < 0.05 was considered statistically significant).

## Results

### Univariate analysis of prognostic factors

The median survival time of all 68 patients was 26 months. The 3- and 5-year OS rates were 20.59% and 5.88%, respectively. Patient age, sex, anatomical sub-region, the degree of differentiation, the presence or absence of cervical lymph node metastasis, smoking status, and alcohol consumption were included in the univariate analysis. No statistically significant differences in OS rates were observed between the two groups (Table 2).

**Table 2:** Results of the univariate analysis of patient prognosis

Variable	Classification	*N*	3-year OS (%)	5-year OS (%)	Median	HR	95% CI	*P* value
Sex	Male	63	23.81	7.47	24	1.142	0.4718–2.765	0.7682
	Female	5	20.00	0.00	32			
Age (years)	≤ 60	50	32.00	0.00	23.5	0.5572	0.2906–1.068	0.0782
	> 60	18	9.14	0.00	24.5			
Subsite	Pyriform sinus	27	22.22	7.41	24	Ps:Pr: 1.1650 Ps:Pw: 0.8446 Pr:Pw: 0.7327	Ps:Pr: 0.6202–2.188 Ps:Pw: 0.4687–1.522 Pr:Pw: 0.3833–1.401	0.6417
	Post-cricoid region	18	22.22	5.56	26			
	Posterior wall	23	26.09	8.70	19			
Differentiation	High	14	21.43	7.14	27	H:M: 0.8906 H:P: 0.7915 M:P: 0.8499	H:M: 0.4698–1.688 H:P: 0.3869–1.619 M:P: 0.4690–1.540	0.7899
	Moderate	35	28.57	11.43	23			
	Poor	19	15.79	5.26	23			
N classification	N0	19	26.32	10.53	24	1.004	0.5760–1.749	0.9899
	N1–3	49	22.45	8.16	24			
Drinking	Yes	42	23.81	7.14	23	1.151	0.6950–1.905	0.5855
	No	26	23.08	11.54	26			
Smoking	Yes	47	21.28	6.38	24	1.181	0.6991–1.994	0.0740
	No	21	28.57	14.29	27			
TPF regime	Yes	35	31.43	8.57	24	0.848	0.5235–1.375	0.5021
	No	33	24.24	6.06	23			
Surgery	Yes	55	27.27	7.27	24	1.044	0.5766–2.055	0.8926
	No	13	30.77	7.69	22			

*CI* confidence interval, *H* high, *HR* hazard ratio, *M* moderate, *OS* overall survival, *P* poor, *Pr* post-cricoid region, *Ps* pyriform sinus, *Pw* posterior wall, *TPF* paclitaxel + platinum + fluorouracil

### OS

#### Intragroup comparisons

**Fig. 1:**
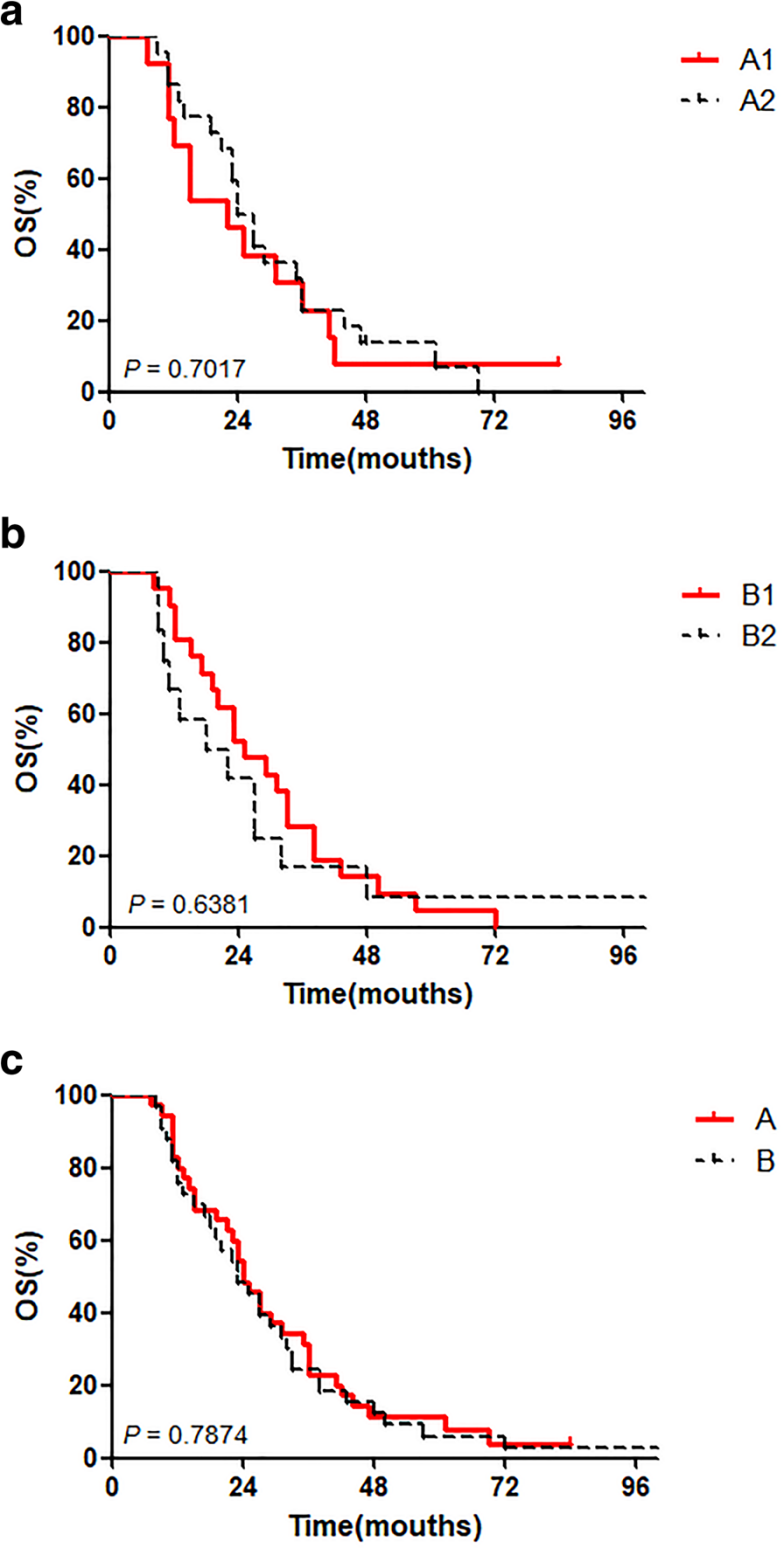
Kaplan–Meier survival curves. **a** The difference in survival rates between groups A1 and A2 as examined using the log-rank test is not statistically significant (*P* = 0.7017). **b** The difference in survival rates between groups B1 and B2 as examined using the log-rank test is not statistically significant (*P* = 0.6381). **c** The difference in survival rates between groups A and B as examined using the log-rank test is not statistically significant (*P* = 0.7874)

The 35 patients in group A were further subdivided into groups A1 and A2 depending on their response to induction chemotherapy. Patients with CR/PR (*N* = 13) were assigned to group A1, and patients with SD (*N* = 22) were assigned to group A2. The 3-year OS rates in groups A1 and A2 were 23.08% and 22.73%, respectively. The 5-year OS rates in groups A1 and A2 were 7.69% and 13.64%, respectively. No statistically significant differences in OS rates were observed between groups A1 and A2 (Fig. 1a).

Among the 33 patients in group B, 21 patients who underwent surgery and post-operative radiotherapy were assigned to group B1, and 12 patients who underwent surgery and post-operative chemoradiotherapy were assigned to group B2. The 3-year OS rates in groups B1 and B2 were 28.57% and 16.67%, respectively. The 5-year OS rates in groups B1 and B2 were 4.76% and 8.33%, respectively. No statistically significant differences in OS rates were observed between groups B1 and B2 (Fig. 1b).

#### Intergroup comparisons

The 3- and 5-year OS rates in group A were 22.86% and 11.43%, respectively, with a median survival of 24 months. The 3- and 5-year OS rates in group B were 24.25% and 6.06%, respectively, with a median survival of 29 months. No statistically significant differences in OS rates were observed between groups A and B (Fig. 1c).

### Laryngeal and oesophageal retention rates

**Fig. 2:**
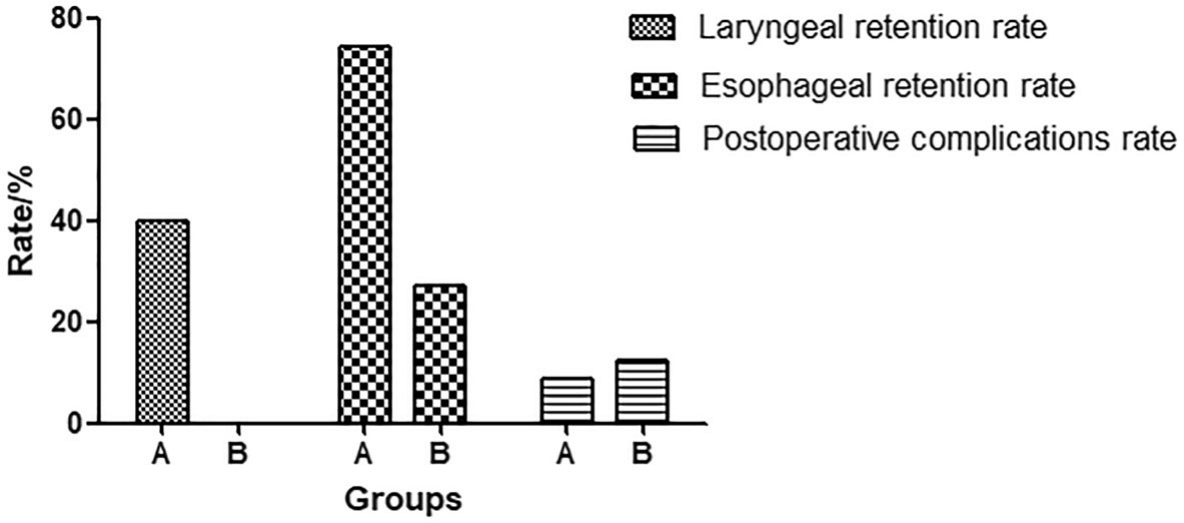
The difference in the laryngeal and the oesophageal retention rates between groups. The difference in the laryngeal and the oesophageal retention rates between groups according to the degree of differentiation is statistically significant (log-rank: *P =* 0.0002 and *P* = 0003, respectively). The difference in postoperative complication rates between groups A and B as examined using the log-rank test is not statistically significant (*P =* 0.9345)

The laryngeal and oesophageal retention rates were significantly higher in group A (40.0% and 74.3%) than in group B (0.0% and 27.3%) (*P* = 0.0001 and *P* = 0.0002, respectively) (Fig. 2).

### Post-operative complications

The most common post-operative complications of advanced hypopharyngeal carcinoma were anastomotic leakage and stricture. In group A, anastomotic leakage occurred in two patients, and anastomotic stricture occurred in one patient, yielding a complication rate of 8.6%. In group B, anastomotic leakage occurred in three patients, and anastomotic stricture occurred in one patient, yielding a complication rate of 12.1%. No statistically significant difference in the incidence of post-operative complications was observed between groups A and B (*P* = 0.9345; Fig. 2).

## Discussion

In this study, we evaluated the potential of induction chemotherapy as an indicator of the management of advanced hypopharyngeal carcinoma with cervical oesophageal invasion. Seventy percent of patients with hypopharyngeal carcinoma are diagnosed at an advanced stage [7]. Advanced hypopharyngeal carcinoma can spread submucosally and tends to invade the cervical oesophagus [9]. Between February 2003 and November 2016, we treated approximately 800 patients who were diagnosed with hypopharyngeal carcinoma, including 75 patients with lesions involving the cervical oesophagus, which accounted for approximately 9.38% of all hypopharyngeal carcinomas. In this study, the 3- and 5-year OS rates of all patients were 20.59% and 5.88%, respectively. These findings were similar to those reported in a previous study of hypopharyngeal and oesophageal carcinoma [5].

Surgery remains the main treatment strategy for advanced hypopharyngeal carcinoma with cervical oesophageal invasion. Surgical treatment mainly comprises primary tumour excision, cervical lymph node dissection, and reconstruction of the pharynx and digestive tract, the latter of which is the biggest challenge. In recent years, there have been several reports of post-operative repair and reconstruction [10, 11]. In this study, reconstruction of the pharynx and digestive tract was primarily performed using one of the three methods described previously [10, 12, 13]. For unilateral pyriform sinus carcinomas where the lesion invades the oesophageal inlet and does not reach the circumference of the cavity of the oesophageal canal, but infiltrates downward for up to 2–3 cm, it is possible to repair the residual hypopharyngeal mucosa and upper oesophageal mucosa using a pectoralis major myocutaneous flap. For large pyriform sinus and posterior pharyngeal wall carcinomas that span the midline with an infiltration depth of 2–3 cm, the digestive tract can be repaired by residual laryngeal anastomosis instead of hypopharyngeal-oesophageal anastomosis. For annular tumours with an infiltration depth of > 2–3 cm from the inlet of the oesophagus, total esophagectomy can be performed, and the oesophagus can be replaced by a gastric lift or free jejunum/colon.

Induction chemotherapy, or neoadjuvant chemotherapy as it is also known, refers to the chemotherapy administered before surgery or radiotherapy, which can reduce the tumour load in a short period of time. Induction chemotherapy has been widely used in clinical practice in recent years. Commonly used regimens include TPF and platinum plus fluorouracil, with the former being significantly more effective than the latter [14]. Induction chemotherapy can reduce the tumour volume and maximise the retention of organ function [15]. It also facilitates screening to determine which patients are likely to respond to treatment with either radiotherapy or concurrent chemoradiotherapy. For unresponsive patients, surgical treatment is preferred. Induction chemotherapy allows for a more individualised, standardised, and precise treatment of advanced hypopharyngeal carcinoma [16].

Our results show that, before 2010, patients were mainly treated surgically, and the treatment methods were relatively simple. With an increase in the number of related studies and advancements in treatment modalities, therapeutic options since 2010 have no longer been dominated by surgery, and adjuvant management methods, such as chemotherapy and radiotherapy, have been used.

The differences in 3- and 5-year OS rates between groups A and B were not statistically significant. However, the laryngeal and oesophageal retention rates were significantly higher in group A than in group B. The laryngeal and oesophageal retention rates are important indicators of the quality of survival in patients with hypopharyngeal and oesophageal carcinoma. We used induction chemotherapy to screen for sensitive patients in whom radiotherapy/concurrent chemoradiotherapy was administered. The OS rate of these patients was not affected. However, their quality of life was improved. Surgical treatment was not delayed in patients unresponsive to induction chemotherapy. In some patients, the tumour size was reduced, which can improve the response to surgery and post-operative adjuvant therapy. This can also serve as an effective treatment. We found that there was no significant difference in the incidence of post-operative complications between the two groups, indicating that induction chemotherapy did not increase the incidence of surgical complications. Surgery is the main treatment for advanced hypopharyngeal carcinoma, and patients who undergo surgery tend to have a longer OS. However, surgical resection is extensive, the post-operative quality of life is poor, and the incidence of post-operative complications is high.

This study has some limitations. First, patients were randomly divided into groups to ensure balance and comparability and to reduce selection bias. Multiple follow-up methods were employed to avoid loss of patients or no response. However, some selection bias (e.g. prevalence-incidence bias) remained. Second, in the univariate analysis, no statistically significant differences in OS rates were observed between the two groups, regardless of age, sex, anatomical sub-region, the degree of differentiation, the presence or absence of cervical lymph node metastasis, smoking status, or alcohol consumption. This could be due to the small number of patients included in this study. Further studies with larger sample sizes are needed to determine the relevance of factors influencing survival.

## Conclusions

The prognosis of advanced hypopharyngeal carcinoma with cervical oesophageal invasion is poor. Induction chemotherapy may be an appropriate first choice to ensure laryngeal and oesophageal preservation in the individualised treatment of advanced hypopharyngeal carcinoma with cervical oesophageal invasion. Questions concerning the choice of induction chemotherapy need to be addressed (e.g. how to determine in advance which patients will respond to induction chemotherapy, whether targeted drugs can be added to improve the efficacy of induction chemotherapy, and how to determine the most appropriate induction chemotherapy regimen). Multicentre collaboration, a large number of case studies, and fundamental research are required.

## Data Availability

The datasets used and/or analysed during the current study are available from the corresponding author on reasonable request.

## Abbreviations

CR: Complete response
CT: Computed tomography
OS: Overall survival
PR: Partial response
SD: Stable disease
TPF: Paclitaxel, platinum, and fluorouracil
